# Analysis of the Thiol Adduction of Linoleate 9,10-Dihydroxy-11*E*‑13-ketones of the Mammalian Skin Barrier and Their Cyclization to Hemiketals

**DOI:** 10.1021/acsomega.5c06050

**Published:** 2025-08-25

**Authors:** Alan R. Brash, William E. Boeglin, M. Wade Calcutt, Markus Voehler, Donald F. Stec, Thomas M. Harris

**Affiliations:** † Department of Pharmacology, Vanderbilt University, Nashville, Tennessee 37232, United States; ‡ Biochemistry, Vanderbilt University, Nashville, Tennessee 37232, United States; § Chemistry, Vanderbilt University, Nashville, Tennessee 37232, United States; ∥ Vanderbilt Institute of Chemical Biology, 5718 Vanderbilt University, Nashville, Tennessee 37232, United States

## Abstract

Enzymic transformations of esterified linoleate (C18:2ω6) in acylceramides help to seal the mammalian skin permeability barrier by promoting Michael addition to protein thiols via the synthesis of 9,10-*trans*-epoxy-11*E*-13-oxo and 9,10-*erythro*-dihydroxy-11*E*-13-oxo oxidation products. In modeling the conjugation of these oxidized linoleates with cysteine and glutathione, we observed anomalous chromatographic and proton NMR results for the thiol conjugates of the dihydroxy-ketones. Adduction eliminates the 11,12 double bond and allows spontaneous C9–C13 cyclization to hemiketal derivatives. This produces a polar diastereomer that chromatographs as interconverting hemiketal species and a less polar diastereomer favored as a single hemiketal and stereochemically pure as indicated by proton NMR. Structural assignments were aided by precedents from carbohydrate research. For the stable diastereomer, COSY analysis revealed a highly unusual 6-bond *J*-coupling between the ring H10 proton and the two H14 protons on the omega-side chain, its detection facilitated by the rigidity of the structure and well-dispersed chemical shifts. Hemiketal formation on protein thiol-adducted oxidized acylceramides in the skin barrier should alter the shape and structure and influence access to enzymes or other proteins. Given the common occurrence of hemiketals and hemiacetals in natural products and synthetic chemistry, the current findings should contribute to their secure structural analysis and characterization.

## Graphical abstract

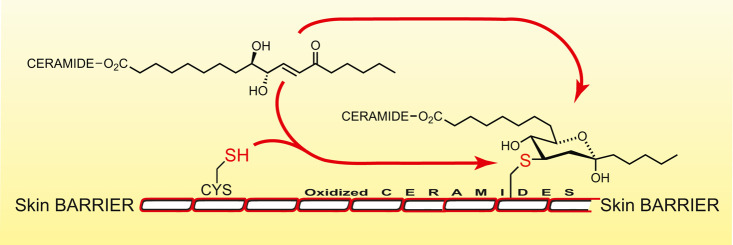

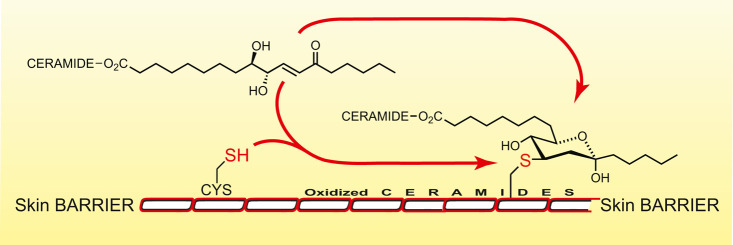

## Introduction

In mammalian skin biology, a complex series of enzymic oxidations facilitate construction of the epidermal water permeability barrier that prevents water loss and allows for life on dry land.^1^ The enzymic substrate is linoleic acid (C18:2ω6) esterified to the omega-hydroxyl of the skin-specific acylceramide Cer-EOS.^2,3^ The linoleate is oxidized by three enzymes acting in series, 12*R*-lipoxygenase, epidermal lipoxygenase-3, and the short-chain dehydrogenase-reductase SDR9C7, producing 9*R*,10*R*-*trans*-epoxy-11*E*-13-oxo-octadecenoate, still esterified in Cer-EOS.^2,4,5^ The structure of the free acid of this oxidized linoleate is shown in Scheme 1, designated as compound **1** and its enantiomer as compound **1a**. The conjugated enone moiety of this linoleate epoxy-ketone is prone to Michael addition, it is highly reactive with thiols,^6,7^ and in the skin, it promotes the covalent binding of the oxidized Cer-EOS to skin barrier protein,^6^ forming an important barrier substructure known as the corneocyte lipid envelope.^8^

**Scheme 1:**
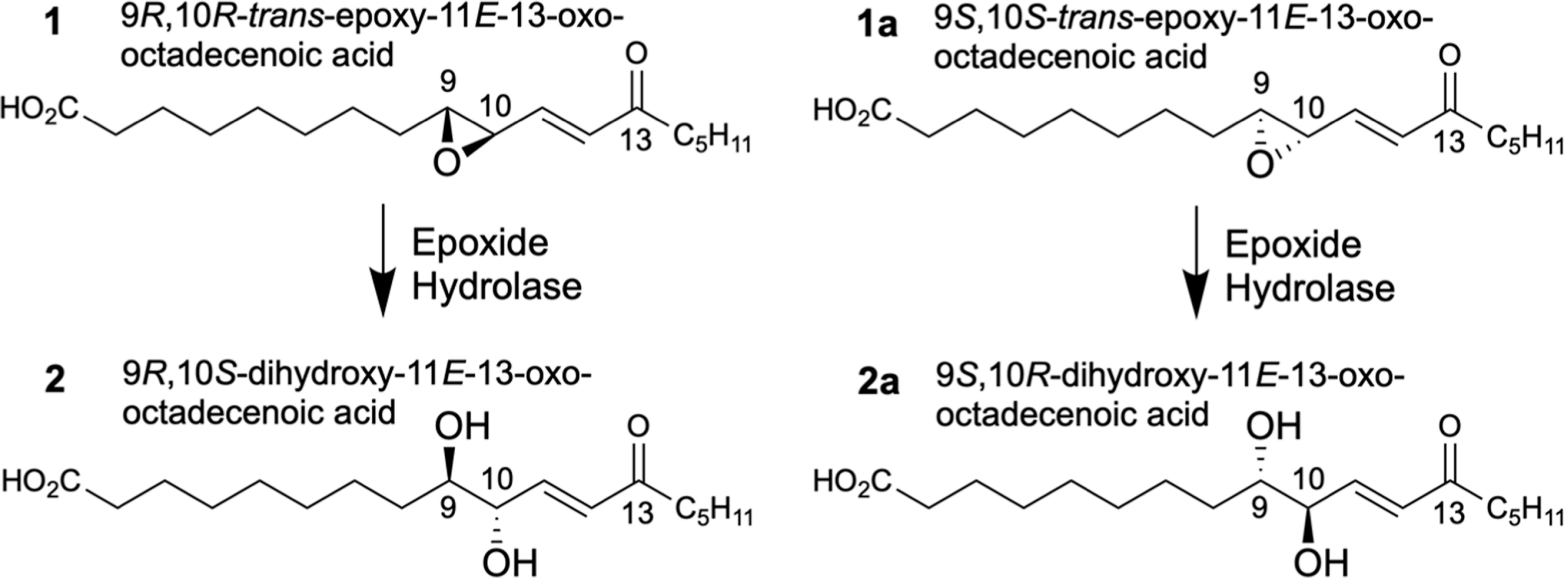


The *trans*-epoxide moiety of **1** is readily transformed by epoxide hydrolases, with reversal of configuration at the 10-carbon, to the corresponding 9,10-dihydroxy-ketone 9*R*,10*S*-dihydroxy-11*E*-13-oxo-octadecenoate shown as compound **2** and its enantiomer as **2a** in Scheme 1.^9^ Recent evidence suggests this enzymatic hydrolysis occurs in construction of the skin permeability barrier such that the covalent binding to protein includes both the epoxy-ketone and dihydroxy-ketone esterified in Cer-EOS.^7,10^ In the course of modeling the thiol conjugation of **1** and **2** by reaction with cysteine or glutathione, we encountered anomalous chromatographic behavior of the thiol conjugates of the dihydroxy-ketones **2** and **2a**. This was matched in the proton NMR analyses, which indicated that the thiol adduction involved loss of the 13-ketone. For either **1** or **2**, the Michael addition of cysteine to the 11-carbon should produce structures with a 9,10-epoxide or diol, 11-sulfide and 13-ketone. This arrangement is evident for the thiol conjugation of the epoxy analogue **1**, while dihydroxy **2** undergoes further rearrangements as the results presented herein establish and is of interest for both technical and perhaps biological considerations.

## Results

### Thiol Adduction of Epoxy-Ketone **1**

Initial NMR experiments to establish the structure and site of glutathione adduction to racemic linoleate 9,10-*trans*-epoxy-11*E*-13-ketone (**1**) were hampered by overlapping signals from four geometric isomers comprised of the two epoxide enantiomers coupled via *R* and *S* configurations of the sulfide. The experiments were repeated using chiral epoxy-ketone and with the diastereomeric *R* and *S* sulfide adducts separated by RP-HPLC. We reported recently that RP-HPLC of cysteinyl adducts of unsaturated fatty acid ketones is improved using an aqueous solvent component of 50 mM potassium phosphate at pH 2.^11^ On account of the acid lability of the epoxide group in **1**, the HPLC solvent was modified here to pH 5.5. Using this solvent system for preparative separations, upon thiol adduction with glutathione, each epoxy-ketone enantiomer gave two well-resolved diastereomers, Figure 1. LC–MS of the cysteine conjugate is illustrated in Supporting Information Figure S1.

**Figure 1:**
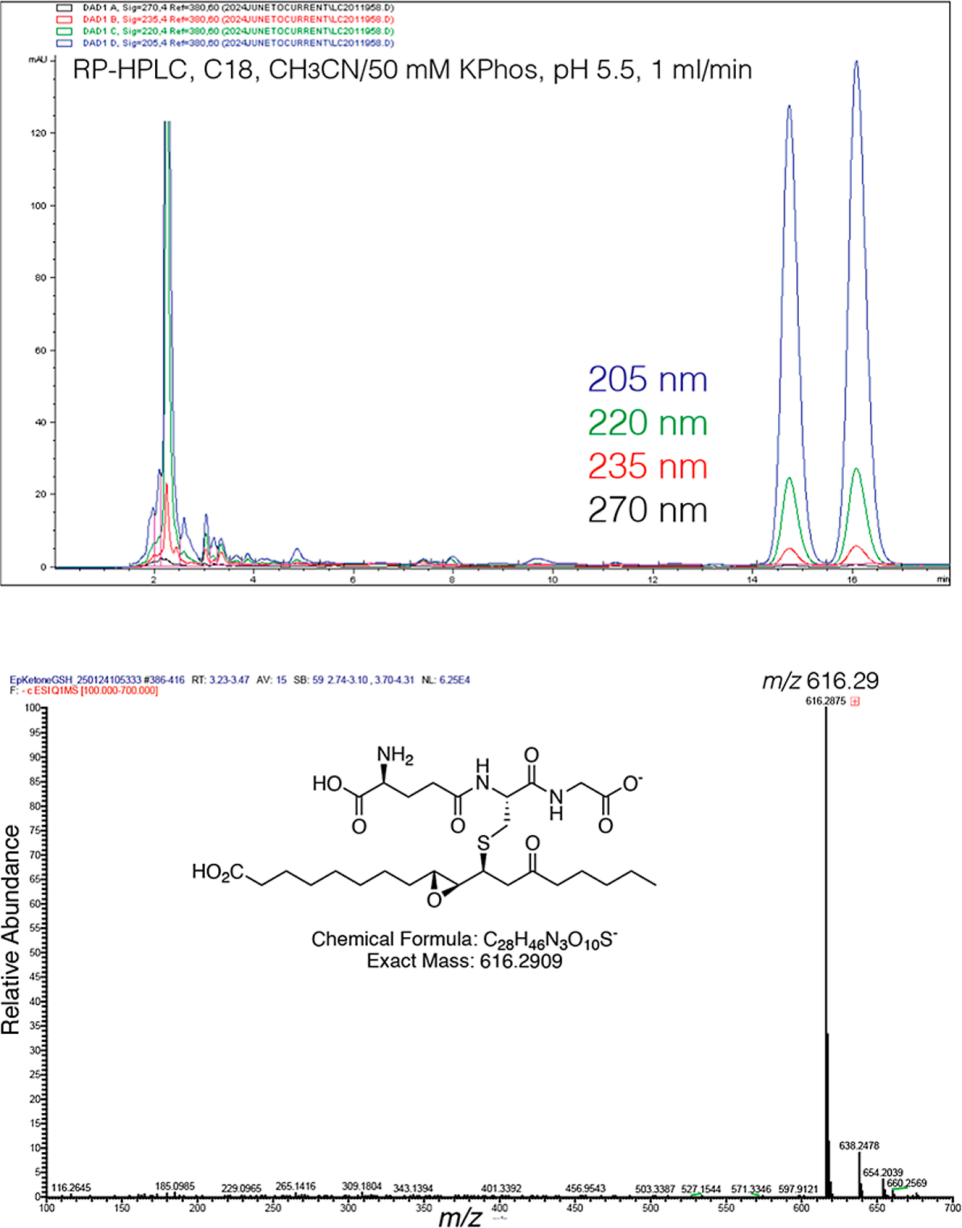
RP-HPLC separation of the two diastereomers of the GSH adducts of **1** (9*R*,10*R*-*trans*-epoxy-11*E*-13-oxo-octadecenoic acid). Top panel: The diastereomers were run on a Waters C18 Symmetry 5 μm column (25 × 0.46 cm) with an isocratic solvent of acetonitrile/aqueous 50 mM potassium phosphate pH 5.5 at a flow rate of 1 mL/min and with diode array UV detection at 205 nm (blue), 220 nm (green), 235 nm (red), and 270 nm (black). Lower panel: Mass spectrum (negative ion ESI) of the second-eluting diastereomer.

^1^H NMR and COSY analysis of the GSH conjugate at 800 MHz gave clear signals for the protons on carbons 9–12 and confirmed adduction at C-11, Supporting Information Figure 2. Complete assignments were aided by HSQC analysis (Figure 2). Of particular relevance for the current study (and to be contrasted with the dihydroxy-ketone) are the chemical shifts of the protons alpha to the 13-ketone. They are located as expected; namely, the H12 protons are well downfield at 2.7 ppm, influenced by both the C11 thiol adduct and the 13-keto functionality, and the H14 methylene is downfield of the fatty acid H2 triplet and overlapping the “**3g**” CH_2_ from the glutathione moiety at 2.35 ppm.

**Figure 2:**
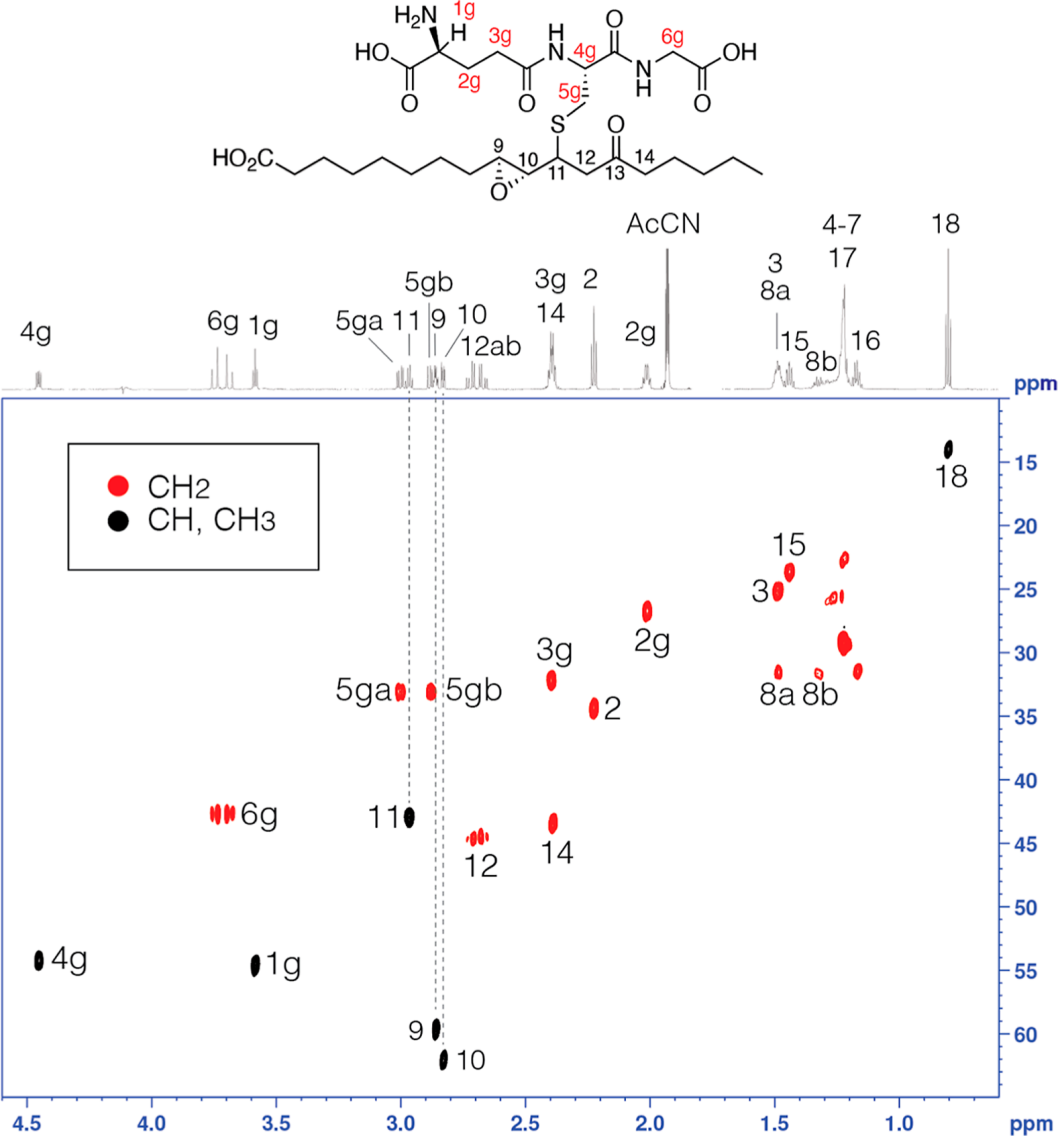
^1^H NMR spectrum (800 MHz) and HSQC analysis of a GSH adduct of **1a** (9*S*,10*S*-*trans*-epoxy-11*E*-13-oxo-octadecenoic acid). The spectrum was recorded on the first-eluting diastereomer from RP-HPLC (cf. Figure 1) in CD_3_CN/D_2_O (60:40 by volume) with water presaturation at 4.1 ppm. Upon thiol adduction, due to the higher priority of sulfur on the 11-carbon, the assignment of H10 changes to 10*R*.

### RP-HPLC and LC–MS of Cysteinyl Adducts of Dihydroxy-ketone **2**

In contrast to the thiol adducts of the parent epoxy-ketone **1**, RP-HPLC and LC–MS of the chiral dihydroxy-ketone **2** produced a relatively complex chromatographic profile, interpreted as an early eluting diastereomer existing as multiple interconverting species and a second less polar diastereomer that elutes as a single well-shaped chromatographic peak. This chromatographic behavior was evident using conventional LC–MS solvents of acetonitrile/10 mM aqueous ammonium acetate for the thiol adducts with glutathione or cysteine. Figure 3 illustrates LC–MS analysis of glutathione adducts of chiral dihydroxyketone **1**, showing a set of early interconverting peaks and one late-eluting and stable isomer. Consistent with this interpretation are the results using racemic dihydroxy-ketone. Racemic dihydroxy-ketone gives an early set of small interconverting peaks and two prominent and later-eluting stable isomers (presumably one from each dihydroxy-ketone enantiomer), illustrated for the cysteine conjugates in Supporting Information Figure S3.

**Figure 3:**
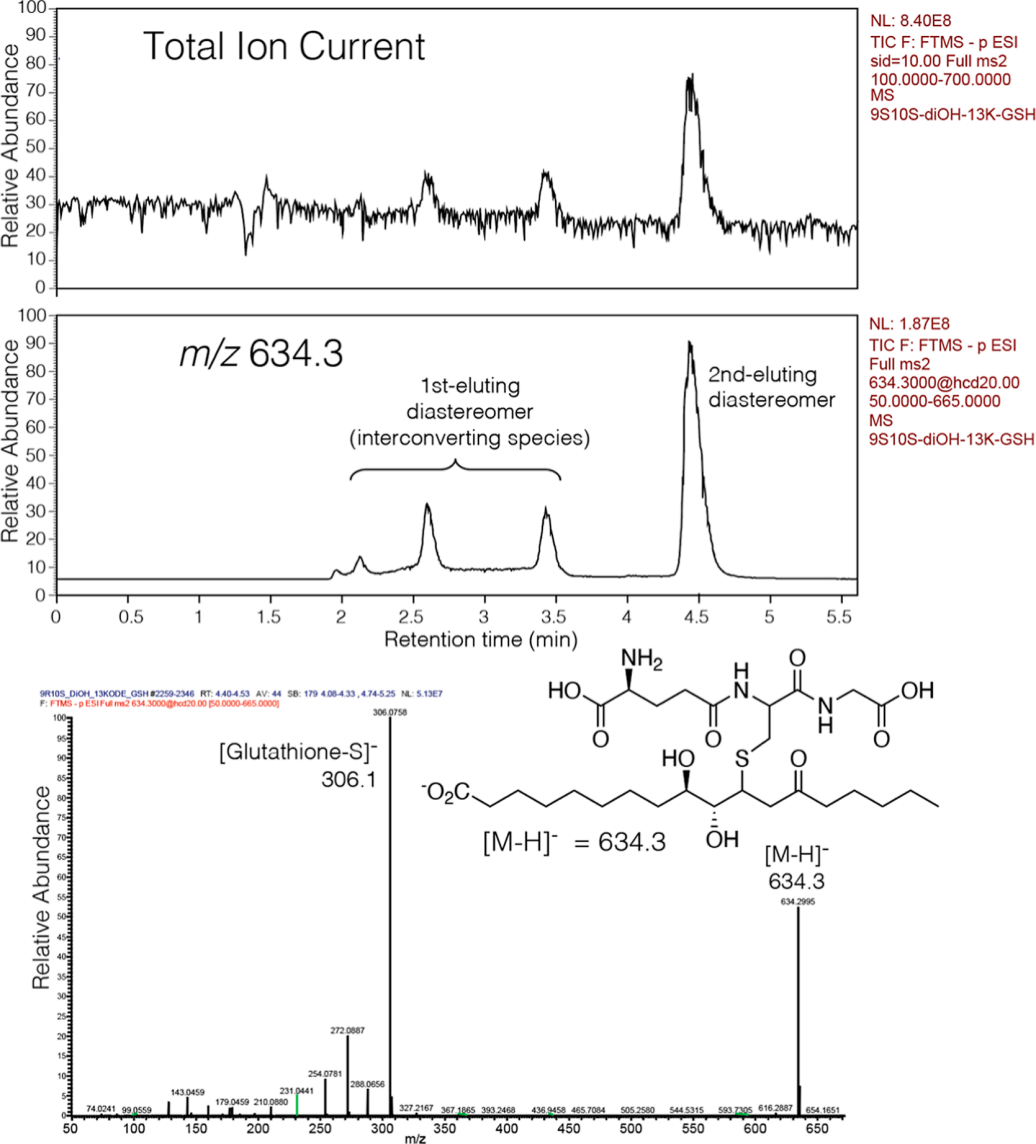
LC–MS analysis of glutathione adducts of **2** (9*R*,10*S*-dihydroxy-11*E*-13-oxo-octadecenoic acid). Top panels: The products were analyzed by LC–MS on a Kinetex 5 μ C18 column (100 × 3 mm) with a solvent of MeOH/H_2_O–10 mM NH_4_Ac, pH 5 at a flow rate of 0.3 mL/min. Adduction of glutathione in the 11*R* or 11*S* configuration gives two diastereomers that differ in their stability. The first-eluting diastereomer appears as two interconverting species as evidenced by the raised baseline between the two peaks at 2.6 and 3.4 min, whereas the second diastereomer chromatographs normally at 4.4 min. (The same chromatographic behavior occurs with the GSH conjugates of **2a**, the enantiomeric 9*S*,10*R*-dihydroxy-11*E*-13-ketone.) Lower panel: Mass spectra were recorded on a Thermo Q Exactive HF hybrid quadrupole/orbitrap scanning in negative ion ESI over a mass range of *m*/*z* 50–650. All isomers gave the same [M – H]^−^ ion; the mass spectrum is of the peak at 4.5 min.

To collect the products for NMR analysis, the HPLC solvent was changed to acetonitrile/50 mM potassium phosphate at pH 2 (1:2, by volume). Using this approach for semipreparative purification, the first diastereomer of the chiral 9,10-*erythro*-dihydroxy-11*E*-13-ketone **2** eluted as a single tailing peak and the second diastereomer well-shaped, as before.

### NMR Analyses of Cysteinyl Adducts of Dihydroxy-ketone **2**

Subsequent NMR analysis of the glutathione adduct of the first-eluting diastereomer from RP-HPLC gave a complex series of multiple small signals in the region of 2–4 ppm that were considered not readily amenable to further analysis. (In retrospect, the spectrum is interpreted as the first-eluting diastereomer in multiple isomeric forms.) The glutathione adducts of the second-eluting diastereomer of the enantiomeric 9*R*,10*S*- and 9*S*,10*R*-dihydroxy-13-ketones **2** and **2a** gave proton NMR spectra interpreted with the aid of COSY and HSQC analyses, Figures 4 and 5 and Supporting Information Figures S4 and S5.

**Figure 4:**
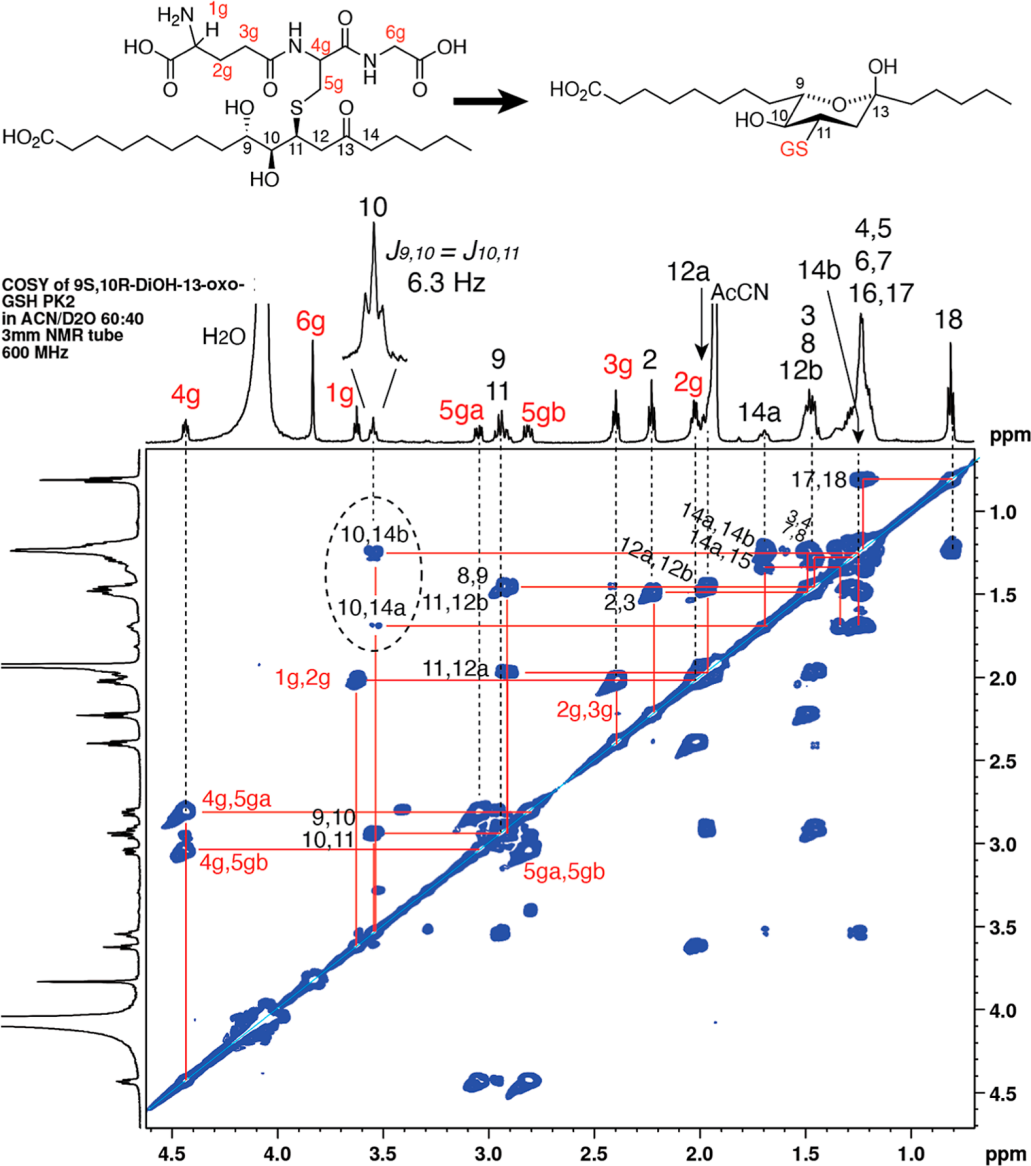
^1^H NMR spectrum (600 MHz) and COSY analyses of the glutathione conjugate of **2a** (9*S*,10*R*-dihydroxy-11*E*-13-keto-octadecanoic acid). The spectrum and COSY of the second-eluting (RP-HPLC) diastereomeric conjugate of 9*S*,10*R*-dihydroxy-11*E*-13-oxo-octadecenoic acid recorded in CD_3_CN/D_2_O (60:40 by volume). Upon thiol adduction, the higher priority of sulfur at C11 changes the C10 hydroxyl assignment to 10*S*. Highlighted and of particular diagnostic value is the triplet of H10 (*J*_9,10_ = *J*_10,11_, 6.3 Hz) and the COSY cross-peaks from H10 to the two H14 protons (Discussion, main text).

**Figure 5:**
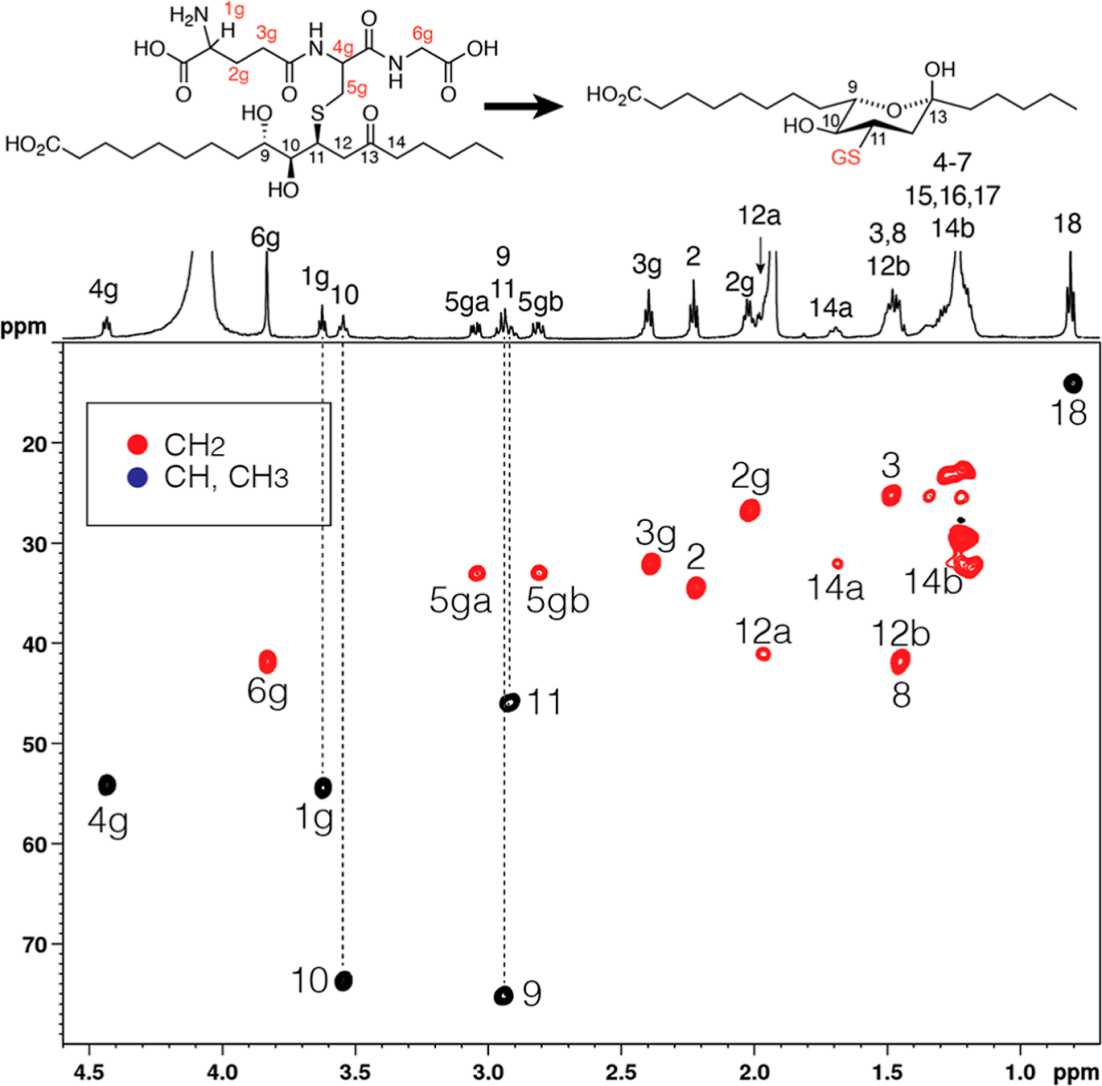
HSQC spectrum of the glutathione conjugate of **2a** (9*S*,10*R*-dihydroxy-11*E*-13-keto-octadecanoic acid). This is the HSQC spectrum of the second-eluting (RP-HPLC) diastereomeric glutathione conjugate of 9*S*,10*R*-dihydroxy-11*E*-13-oxo-octadecenoic acid recorded in CD_3_CN/D_2_O (60:40 by volume). The COSY spectrum is shown in Figure 4. The data indicate the original 13-ketone is not present and the linear structure (left side above) has cyclized to the hemiketal shown on the right.

As expected, the protons from the glutathione moiety were similar in their chemical shifts to the epoxy-ketone-GSH adducts, as were many of the protons from the fatty acid carbon chain with the vicinal hydroxy protons at 2.92 and 3.55 ppm. Strikingly, however, the H12 and H14 protons were absent from the locations established for the parent epoxy-ketones and instead are located well upfield. The COSY cross-peaks from H11 and the pair of signals on HSQC locate the two H12 protons at 1.47 and 2.02 ppm in both the 9*R*,10*S* and 9*S*,10*R* enantiomers. The H14 protons are also split (found at 1.25 and 1.70 ppm), and the COSY analysis reveals a remarkable long-range W-coupling (more akin to WW) with H10, Figure 4. For this long-range *J*-coupling to be feasible, a rigid structure is required, typically involving a ring.^12–16^ These findings on H12 and H14, contrasting with the thiol adducts of the epoxy-ketone, all point to the 13-ketone being absent in the structure. Notably, coupling of the thiol at C11 eliminates the 11,12-*trans* double bond, allowing hemiketal formation via reaction of a dihydroxy hydroxyl on the fatty acid chain with the 13-ketone. By analogy to hemiacetal formation on glucose, which strongly favors the 6-membered ring (and not 5-membered), similar steric considerations upon thiol adduction to the 9,10-dihydroxy-11*E*-13-ketone support involvement of the C9-hydroxyl and formation of a hemiketal with a 6-membered ring, Figure 6. This leaves the 10-hydroxyl on the ring with its axial vicinal proton available for long-range coupling to the two protons of H14.

**Figure 6:**
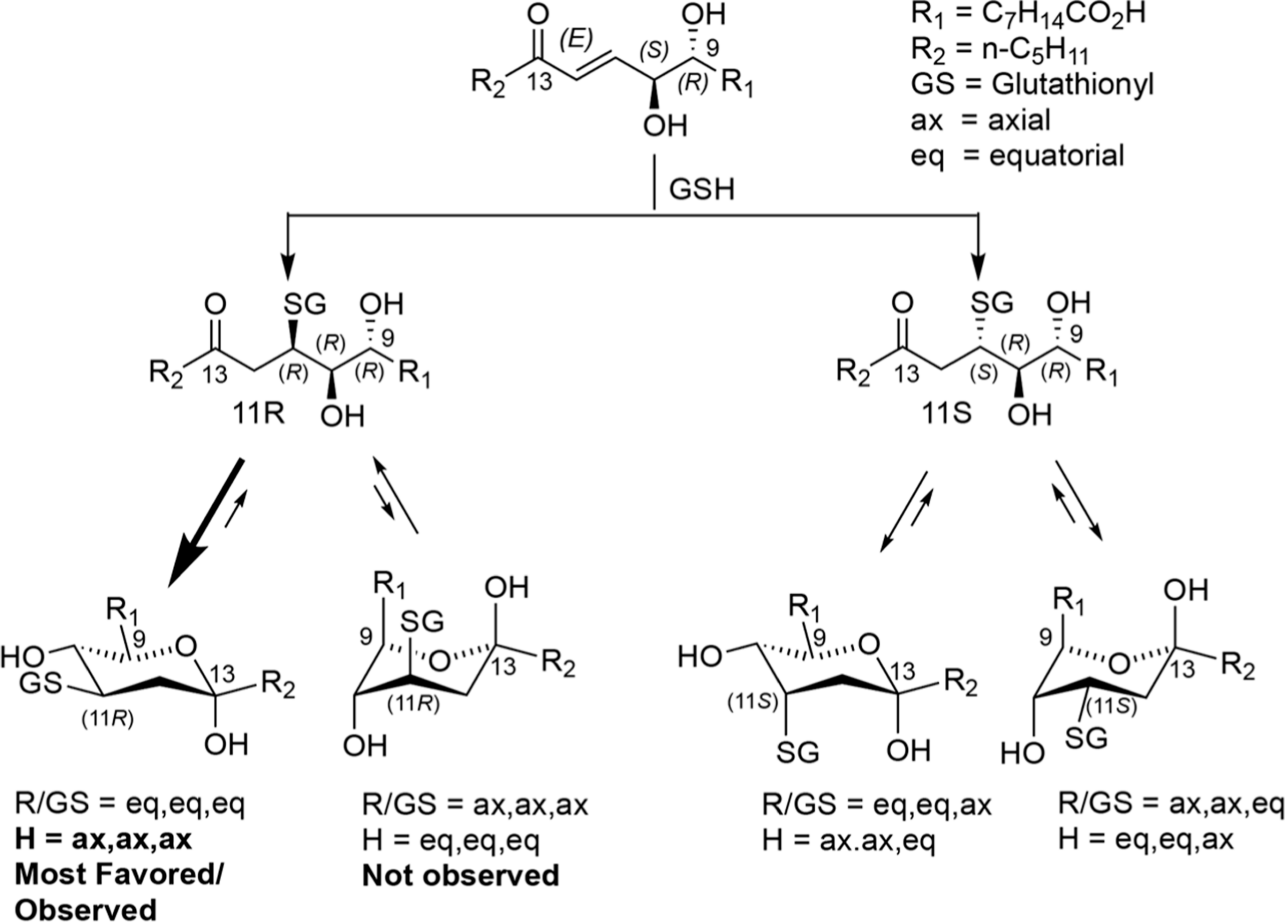
Adduction of dihydroxy-ketones with glutathione and their cyclization to hemiketals. The naturally occurring dihydroxy-ketone, **2**, has 11*R* and 11*S* adducts with glutathione (GSH). The 11*S* adducts (right-hand side and that appear as multiple peaks on RP-HPLC) exist as interconverting hemiketals. The most favored form of the 11*R* isomer (right-hand structure) is the late-eluting and “stable” diastereomer on RP-HPLC and is deduced to have H9,10,11 in the axial/axial/axial configurations (Discussion, main text).

LC–MS of the glutathione adducts can now be interpreted as the chromatography of isomeric hemiketals (all identical in mass to the acyclic structure). Glutathione reacts with a chiral 9,10-dihydroxy-13-ketone to produce two diastereomers, epimeric at C11. On reversed-phase LC–MS, the first-eluting diastereomer appears mainly as two interconverting species of similar abundance (two interconverting hemiketals), the second as a conventional chromatographic peak (a stable, and as judged by NMR, stereochemically pure hemiketal), Figures 3–5.

## Discussion

The results outline what initially was a perplexing series of LC–MS and ^1^H NMR findings on the thiol adduction of 9,10-dihydroxy-11*E*-13-oxo linoleates (**2**) and solved their occurrence as hemiketals. The facility to cyclize to hemiketal applies to any adduction or saturation that removes the 11,12 double bond, allowing the interaction of C9–C13. With adduction on the 11-carbon, each 9,10-dihydroxy-11*E*-13-keto enantiomer gives a pair of diastereomers, a more polar on RP-HPLC existing as interconverting species (presumably the open-chain form allowing interconversion of hemiketals differing in axial or equatorial configurations), and the second-eluting diastereomer existing with one configuration strongly favored and appearing as stereochemically pure on NMR. Insight on the structure of the “stable” diastereomer comes from the coupling constants *J*_9,10_ and *J*_10,11_ being equal, with H10 appearing as a triplet (*J*_9,10_ = *J*_10,11_ = 6.3 Hz), as observed in the spectrum of the late-eluting diastereomer of both enantiomers (Figure 4 and Supporting Information Figure S4). Based on the anomeric effect established for carbohydrates,^17,18^ the anomeric hydroxyls are assigned as axial. It also follows that equatorial protons have much smaller couplings with either axial or equatorial protons on adjacent carbon atoms. The relatively high value of 6.3 Hz for the *J*-coupling to H10 is most compatible with H9, H10, and H11 all being axial. Only one of the four possible hemiketal configurations formed by thiol adduction to the naturally occurring enantiomer **2** has the axial/axial/axial arrangement of the H9–H10–H11 protons, as shown in Figure 6. This in turn dictates that the “stable” conjugate has glutathione adducted in the 11*R* configuration (Figure 6, left-hand structure). By similar reasoning, the second-eluting and “stable” conjugate of enantiomer **2a** with axial/axial/axial H9–H10–H11 has GSH adducted in the 11*S* configuration (Supporting Information Figure 6).

An interesting technical issue from the ^1^H NMR analyses is the exceptional long-range 6-bond *J*-coupling between the C10 axial proton and the two H14s (indicated in red in Figure 7). This is uncommon and goes beyond the typical W-coupling.^12^ Nonetheless, longer-range couplings (^5^ *J* and higher) are reported, particularly in acetylenes and allenes.^13,16^ In the present case, the coupling is observed in no small part because the chemical shifts are dispersed. This avoids virtual coupling, which would cause broadening of signals and obscuring of the long-range interaction. Rigidity of the structure is required for long-range spin–spin coupling; however, it is not the whole explanation. Sterols are highly rigid structures, yet as far as we are aware, there are no reported sterol spectra exhibiting this phenomenon. In addition to the interest as an observation in proton NMR, the occurrence of this long-range coupling further consolidates the existence of the thiol adducts as cyclized species, as this ^6^ *J* spin–spin coupling would not be possible without the structure the 6-membered ring provides.

**Figure 7:**
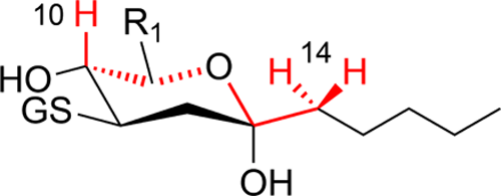
Long-range ^6^ *J*-coupling in the proton NMR spectra of thiol adducts of dihydroxy-ketones. The 6-bond coupling of H10 and the two H14 protons is illustrated in red.

The linoleate 9,10-epoxy- and 9,10-dihydroxy-11*E*-13-ketones are important constituents of the mammalian skin water permeability barrier. Their rates of reaction with thiols are 100-fold higher than for a simple unsaturated fatty acid ketone,^7^ and it has become clear that the whole rationale for their biosynthesis in the outer epidermis is to promote the covalent attachment to thiol residues in the barrier proteins. Based on our results, the thiol adducts with the dihydroxy derivatives will exist mainly as cyclic hemiketals in the skin barrier. It is an open question as to whether this change in shape and structure could influence access to enzymes or other proteins. It is also a point for consideration that the thiol adduction of oxidized Cer-EOS in the epidermis is a reversible covalent attachment^2,6,7^ and, as found herein for one diastereomer of the thiol adducts of the dihydroxy-ketone, perhaps the instability of half of the configurations contributes to the availability of the adducts for technical analysis. More generally, at least one other instance of hemiketal formation upon glutathione adduction is reported,^19^ and the interaction has also found an application in a method for the sensitive detection of cellular thiols.^20^ Given the common occurrence of hemiketals and hemiacetals in natural products and synthetic chemistry, the current findings should contribute to their secure structural analysis and characterization.

## Methods

### Preparation of 9,10-*trans*-Epoxy-11*E*-13-oxo-octadecenoic acid (**1**)

The epoxy-ketone **1** was prepared as described via transformation of linoleic acid with soybean lipoxygenase followed by oxidation of the 9*cis*,11*trans*-13-hydroxy product, its isomerization to 9*trans*,11*trans*-13-hydroxy, and oxidation to the 13-oxo, and epoxidation to the racemic epoxy-ketone.^7^ The enantiomers were resolved as described on a semipreparative scale (300 μg per injection) using a Lux 5 μm Amylose-1 column (25 × 1 cm), with 9*S*,10*S*-*trans*-epoxy-11*E*-13-oxo-octadecanoic acid eluting before the 9*R*,10*R* enantiomer.^7^ 9,10-*trans*-Epoxy-11*E*-13-oxo-octadecenoate methyl ester. C_6_D_6_, δ: 6.45 (1H, dd, *J*_10,11_ = 7.0, *J*_11,12_ = 15.9 Hz, H11); 6.235 (1H, d, *J*_11,12_ = 15.8 Hz, H12); 3.355 (3H, s, CH_3_O; 2.80, 1H, dd, *J*_9,10_ = 1.5 Hz, *J*_10,11_ = 6.9 Hz, H10); 2.499 (1H, dt, *J*_9,10_ = 1.9 Hz, *J*_8,9_ = 5.5 Hz, H9); 2.157 (2H, t, *J* = 7.3 Hz, H14); 2.109 (2H, t, *J* = 7.4 Hz, H2); 1.55 (4H, m, H3 and H15); 1.29 (2H, m, H8); 1.23–1.09 (12H, m, H4, 5, 6, 7, 16, 17); 0.835 (3H, t, *J* = 7.2 Hz, H18). The NMR spectrum is illustrated in Noguchi et al., and the epoxy-ketone was quantified based on UV absorbance, λ_max_ 234 nm in EtOH, ε = 12,500 M^–1^ cm^–1^.^21^

### Preparation of 9,10-*erythro*-Dihydroxy-11*E*-13-oxo-octadecenoic acid (**2**)

This was prepared from the parent epoxy-ketone using recombinant human soluble epoxide hydrolase.^7^ Resolution of the 9,10-dihydroxy-*erythro*-11*E*-13-oxo-octadecenoic acids on a semipreparative scale (300 μg injections) used a reversed-phase chiral column, Chiralpak AD-RH (15 × 0.46 cm, 5 μm, with guard column, 1 × 0.4 cm, 5 μm) and a solvent of acetonitrile/water/glacial acetic acid (60:40:0.01 by volume) at a flow rate of 0.5 mL/min with retention times of 8.2 min (9*R*,10*S* enantiomer, **2**) and 13.2 min (9*S*,10*R*, **2a**). 9*R*,10*S*-Dihydroxy-11*E*-13-oxo-octadecenoate methyl ester, C_6_D_6_, δ 6.77 (1H, dd, *J*_11,12_ = 15.9, *J*_10,11_ = 5.0 Hz, H11); 6.33 (1H, dd, *J*_11,12_ = 15.9 Hz, *J*_10,12_ = 1.7 Hz, H12); 3.83 (1H, m, H10); 3.35, (3H, s, OCH_3_); 3.33, 1H, m, H9); 2.24, (2H, t, *J* = 7.3 Hz, H14); 2.10, (2H, t, *J* = 7.4 Hz, H2); 1.71 (weak, <1H, m, H9–OH); 1.60 (3H, m, H15, H10-OH); 1.53, (2H, p, H3); 1.35–1.22, (2H, m, H8); 1.22–1.05 (10H, H17, 4, 5, 6, 16); 0.83, (3H, t, H18). The dihydroxy-ketone was quantified based on UV absorbance, λ_max_ 226.5 nm in EtOH, ε = 12,500 M^–1^ cm^–1^ and the mass spectrum of the methyl ester methoxime trimethylsilyl ether is illustrated in Noguchi et al.^21^

### Preparation and Purification of Thiol Adducts

Reactions with 25–100 μg/mL linoleate conjugated enones with cysteine or glutathione were conducted in 0.1 M potassium phosphate (pH 7.5) and monitored by UV spectroscopy (disappearance of the conjugated enone chromophore) by repetitive scanning over 350–200 nm using a Cary 60 UV–vis spectrometer (Agilent). The solution was adjusted to pH 5 and extracted on a 30 or 60 mg Oasis cartridge (Waters) with elution of product using acetonitrile/water (70:30 by volume).

NMR analysis of a GSH adduct of 9*R*,10*S*-dihydroxy-11*E*-13-oxo-octadecenoic acid (AD-RH chiral column peak 1, RP-HPLC peak 2): (the cysteine and GSH adducts have the 9*R*,10*R* configuration because the higher priority of sulfur at C11 switches 10*S* to 10*R*; chirality at C11 is not determined). ^1^H NMR (600 MHz, CD_3_CN/D_2_O, 60:40 by volume) chemical shift, number of protons, multiplicity, *J*, and proton number, using “g” designation for protons in glutathione (see Figures): δ (ppm), 4.467 (1H, dd, *J* = 5.9, 8.0 Hz, 4g); 3.824 (2H, s, 6g); 3.621 (1H, t, *J* = 6.3 Hz, 1g); 3.550 (1H, br. t, *J* = approximately 7 Hz, H10); 4.467 (1H, dd, *J* = 5.01, 8.37 Hz, 4g); 3.621 (1H, t, *J* = 6.36 Hz, 1g); 3.548 (1H, t, *J* = 6.8 Hz, H10); 2.82–2.98 (4H, m, 5g­(a,b), H9, and H11); 2.406 (2H, t, *J* = 7.4 Hz, 3g); 2.229 (2H, t, *J* = 7.5 Hz, H2); 2.03 (3H, m, 2g, 12a); 1.93 CH_3_CN solvent; 1.70 (1H, br. t, *J* = approximately 11 Hz, H14a); 1.4–1.55 (5H, m, H3, H8, H14b); 1.2–1.35 (15H, m, H4–7, H14b, and H15–17); 0.814 (3H, t, *J* = 7.1 Hz, H18).

^1^H NMR analysis of a glutathione conjugate of 9*S*,10*R*-dihydroxy-11*E*-13-oxo-octadecenoic acid (AD-RH chiral column peak 2, RP peak 2, of chirality 9*S*,10*S* in the glutathione adduct, and C11 chirality not determined). ^1^H NMR (600 MHz, CD_3_CN/D_2_O, 60:40 by volume) chemical shift, number of protons, multiplicity, *J*, and proton number, using “g” designation for protons in glutathione (see Figures): δ (ppm), 4.433 (1H, dd, *J* = 5.9, 8.0 Hz, 4g); 3.833 (2H, s, 6g); 3.625 (1H, t, *J* = 6.3 Hz, 1g); 3.546 (1H, t, *J* = 8.8 Hz, H10); 3.049 (1H, dd, *J* = 5.6, 13.8 Hz, 5ga); 2.90–2.97 (2H, m, H9, H11); 2.815 (1H, dd, 8.4, 13.6 Hz, 5gb); 2.397 (2H, t, *J* = 7.2 Hz, 3g); 2.228 (2H, t, *J* = 7.5 Hz, H2); 2.011 (2H, q, *J* = Hz, 2g); 1.98 (1H, shoulder of CH_3_CN solvent, H12a); 1.93 CH_3_CN solvent; 1.70 (1H, br. t, *J* = approximately 11 Hz, H14a); 1.45–1.55 (5H, m, H3, H8, H12b); 1.15–1.35 (15H, m, H4–7, H14b, and H15–17); 0.812 (3H, t, *J* = 7.1 Hz, H18).

The above spectra were recorded on the GSH diastereomer eluting as reversed-phase peak 2. For both of the 9,10-*erythro*-dihydroxy-11*E*-13-keto enantiomers, RP peak 1 gave more complex proton NMR spectra, with many apparent signals of relative 0.5 intensity, probably accounted for by an equilibrium mixture of two configurations of hemiketal, as noted in the main text.

### LC–MS Methods

LC–MS analyses used a TSQ Vantage triple quadrupole mass spectrometer for product identification and quantitative analyses and a Thermo Q Exactive HF hybrid quadrupole/orbitrap for high-resolution MS. The TSQ Vantage triple quadrupole mass spectrometer (Thermo Scientific, San Jose, CA) is equipped with an Ion Max API source, a HESI-II probe, and a 50 μm ID stainless steel high-voltage capillary. Data acquisition and analysis were carried out using Xcalibur v.2.1.0, Vantage v.2.3.0, and LCQuan v.2.7.0 software (Thermo). A Kinetex C18 analytical column (100 × 3 mm), 2.6 mm particle size, (Phenomenex, Torrance, CA) was used for all chromatographic separations with solvent indicated in the text, typically acetonitrile/aqueous 10 mM NH_4_Ac, pH 5 in 50:50 proportions at flow rates of 0.5 mL/min.

### NMR Analyses

^1^H NMR and ^1^H,^1^H COSY NMR experiments were acquired using a 14.0 T Bruker magnet equipped with a Bruker AV-III console operating at 600.13 MHz or using a Bruker 800 MHz spectrometer. All spectra were acquired in 3 mm NMR tubes using a Bruker 5 mm TCI cryogenically cooled NMR probe. Chemical shifts were referenced internally to the middle acetonitrile peak at 1.93 ppm in CD_3_CN/D_2_O (60:40 by volume) or benzene-*d*_6_ (7.16 ppm). Glutathione adducts were recorded in *d*_4_-methanol or dissolved in a 60:40 mixture of CD_3_CN/D_2_O.

## Supplementary Material



## Data Availability

All data will be made available upon reasonable request.

## Notes

Figures including LC–MS analysis of adducts of 9*R*,10*R*-*trans*-epoxy-11*E*-13-oxo-octadecenoic acid with L-cysteine, partial ^1^H NMR spectrum and COSY analyses of a glutathione conjugate of **1a** (9*S*,10*S*-*trans*-epoxy-11*E*-13-ketone), LC–MS analysis of cysteine adducts of racemic 9,10-*erythro*-dihydroxy-11*E*-13-oxo-octadecenoic acid, ^1^H NMR spectrum (600 MHz) and COSY analyses of the glutathione conjugate of **2** (9*R*,10*S*-dihydroxy-11*E*-13-oxo-octadecanoic acid), HSQC spectrum of the glutathione conjugate of **2** (9*R*,10*S*-dihydroxy-11*E*-13-oxo-octadecanoic acid), and hemiketal formation upon glutathione adduction to 9*S*,10*R*-dihydroxy-ketone **2a** (PDF)

## Abbreviations

Cer-EOS: ceramide-esterified-omega-hydroxy-sphingosine
COSY: correlation spectroscopy
ESI: electrospray ionization
GSH: glutathione
HSQC: heteronuclear single quantum coherence
LC–MS: liquid chromatography–mass spectrometry
NP-HPLC: normal-phase high-pressure liquid chromatography
RP-HPLC: reversed-phase high-pressure liquid chromatography
SDR9C7: short-chain dehydrogenase-reductase family 9C member 7
UV: ultraviolet
