# Supplementary material for: Analysis of the Thiol Adduction of Linoleate 9,10-Dihydroxy-11E‑13-ketones of the Mammalian Skin Barrier and Their Cyclization to Hemiketals
Source: ACS Omega. 2025 Aug 25;10(35):40512–20. doi: 10.1021/acsomega.5c06050 (PMC12423870; doi:10.1021/acsomega.5c06050)
Supplement: Supplementary file 1 [file ao5c06050_si_001.pdf]

Supporting Information

Analysis of the thiol adduction of linoleate 9,10-dihydroxy-11*E*-13-ketones of the mammalian skin barrier and their cyclization to hemiketals

Alan R. Brash[1,4,*], William E. Boeglin[1], M. Wade Calcutt[2], Markus Voehler[3], Donald F. Stec[3], and Thomas M. Harris[3,4]

Departments of Pharmacology[1], Biochemistry[2], Chemistry[3] and the Vanderbilt Institute of Chemical Biology[4], Vanderbilt University, Nashville, TN, USA

* Corresponding author, Alan R Brash: alan.brash@vanderbilt.edu

Analysis of the thiol adduction of linoleate 9,10-dihydroxy-11*E*-13-ketones of the mammalian skin barrier and their cyclization to hemiketals

Alan R. Brash, William E. Boeglin, M. Wade Calcutt, Markus Voehler, Donald F. Stec, and Thomas M. Harris

Supplemental Figure S1: LC-MS analysis of adducts of 9*R*,10*R*-*trans*-epoxy-11*E*-13-oxo-octadecenoic acid with L-cysteine

The adducts were chromatographed on a Kinetex 2.6 µm C18 column (100 x 3 mm) with a solvent of MeOH/H_2O-10 mM NH_4Ac pH 5.0 (60:40, by volume) at a flow rate of 0.3 ml/min. Mass spectra were recorded on a TSQ Vantage scanning in ESI with alternate positive and negative ionization over a mass range of *m/z* 100 – 700. On the Thermo Vantage, masses are accurate to one decimal place. The two epoxy-ketone-Cys diastereomers elute at 4.5 and 4.8 min with similar mass spectra (illustrated for the first-eluting diastereomer).

Supplemental Figure S2: Partial ^{1}H-NMR spectrum and COSY analyses of a glutathione conjugate of 1a (9*S*,10*S*-*trans*-epoxy-11*E*-13-ketone)

Partial 800 MHz spectrum (1.1 – 3.3 ppm) and COSY of the first-eluting (RP-HPLC) diastereomeric conjugate of 9*S*,10*S*-*trans*-epoxy-11*E*-13-oxo-octadecenoate with glutathione recorded in CD_3CN/D_2O (60:40 by volume). Thiol adduction at C11 of the epoxy-ketone is clearly evidenced. Not included in this partial spectrum are protons from the glutathione moiety further downfield: 1g, a triplet at 3.58 ppm; 6g(ab), two doublets centered on 3.72 ppm; and the 4g, a double of doublets at 4.46 ppm; also not on this partial spectrum, the terminal methyl triplet at 0.82 ppm. The full 1D spectrum is illustrated in the main text in Figure 2.

Supplemental Figure S3: LC-MS analysis of cysteine adducts of racemic 9,10-*erythro*-dihydroxy-11*E*-13-oxo-octadecenoic acid

L-Cysteine adducts to racemic dihydroxy-ketone in the 11*R* and 11*S* configuration on each enantiomer, giving four products in total. The two prominent peaks at 7.4 and 8.1 min are single stable species of adduct, one from each enantiomer, that chromatograph normally. The early-eluting diastereomers from each enantiomer are a mixture of interconverting species eluting at retention times between 3 and 6 minutes. (Similar chromatographic behavior is illustrated for the GSH adducts of a chiral dihydroxy-ketone in the main text, Figure 3). Top panel: Total Ion Current. Middle: profile of negative ion *m/z* 448, M-H$^-$. Lower panel: mass spectrum of the peak at retention time 8.1 min (*m/z* 448.23, M-H$^-$). The products were analyzed by LC-MS on a Kinetex 2.6 µ C18 column (100 x 3 mm) with a solvent of MeOH/H_2O-10 mM NH_4Ac, pH 5 (60:40, by volume) at a flow rate of 0.3 ml/min. Mass spectra were recorded on a Thermo Q Exactive HF hybrid quadrupole/orbitrap scanning in negative ion ESI over a mass range of m/z 100 – 700.

Supplemental Figure S4: ^{1}H-NMR spectrum (600 MHz) and COSY analyses of the glutathione conjugate of 2 (9*R*,10*S*-dihydroxy-11*E*-13-oxo-octadecanoic acid)

The spectrum and COSY of the second-eluting (RP-HPLC) diastereomeric conjugate of 9*R*,10*S*-dihydroxy-11*E*-13-oxo-octadecenoic acid recorded in CD_3CN/D_2O (60:40 by volume). Upon thiol adduction the higher priority of sulfur at C11 changes the C10 hydroxyl assignment to 10*S*. The main difference from the spectrum the GSH conjugate of the enantiomeric dihydroxy-ketone is the signals from 5g(ab), H9 and H11 are a single cluster in the above spectrum (cf. Figure 4, main text). GS = Glutathione.

Supplemental Figure S5: HSQC spectrum of the glutathione conjugate of 2 (9*R*,10*S*-dihydroxy-11*E*-13-oxo-octadecanoic acid)

This is the HSQC spectrum of the second-eluting (RP-HPLC) diastereomeric glutathione conjugate of **2**, 9*R*,10*S*-dihydroxy-11*E*-13-oxo-octadecenoic acid, recorded in CD_3CN/D_2O (60:40 by volume). The COSY spectrum is shown in Supplemental Figure S4.

Supplemental Figure 6: Hemiketal formation upon glutathione adduction to 9*S*,10*R*-dihydroxy-ketone 2a

The 11*R* adducts (left hand side, and that appear as multiple peaks on RP-HPLC) exist as interconverting hemiketals. The most favored form of the 11*S* isomer (right hand structure) is the late-eluting and "stable" diastereomer on RP-HPLC and is deduced to have H9-H10-H11 in the axial/axial/axial configurations (Discussion, main text).

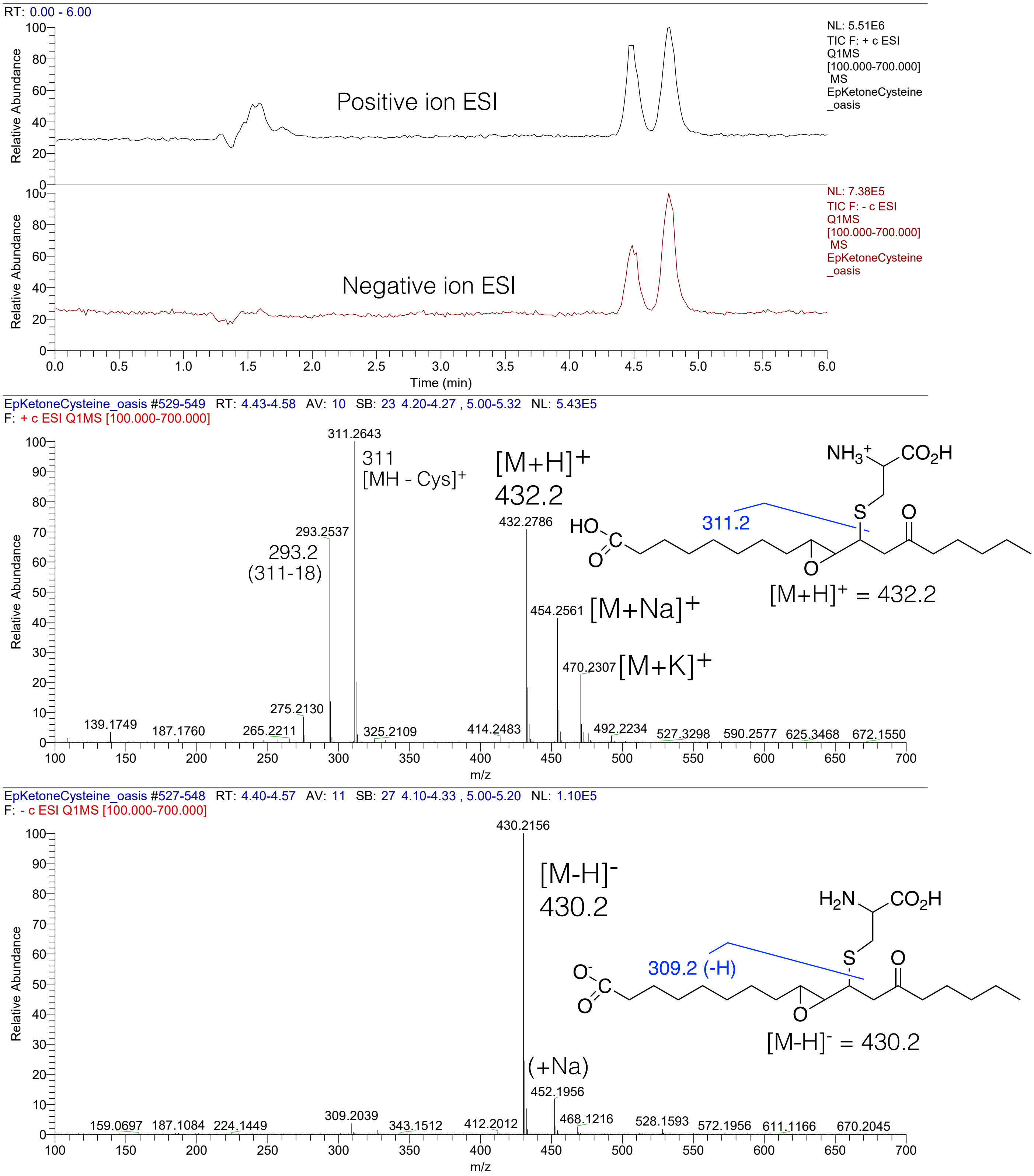

Supplemental Figure S1: LC-MS analysis of adducts of 9*R*,10*R*-trans-epoxy-11*E*-13-oxo- octadecenoic acid with L-Cysteine

The adducts were chroma tographed on a Kinetex 5 µm C18 column (100 x 3 mm) with a solvent of MeOH/H_2O-10 mM NH_4Ac, (60:40) pH 5.0 at a flow rate of 0.3 ml/min. Mass spectra were recorded on a TSQ Vantage scanning in ESI with alternate positive and negative ionization over a mass range of m/z 100 – 700. On the Thermo Vantage, masses are accurate to one decimal place. The two epoxy-ketone-Cys diastereomers elute at 4.5 and 4.8 min with similar mass spectra (illustrated for the first-eluting diastereomer).

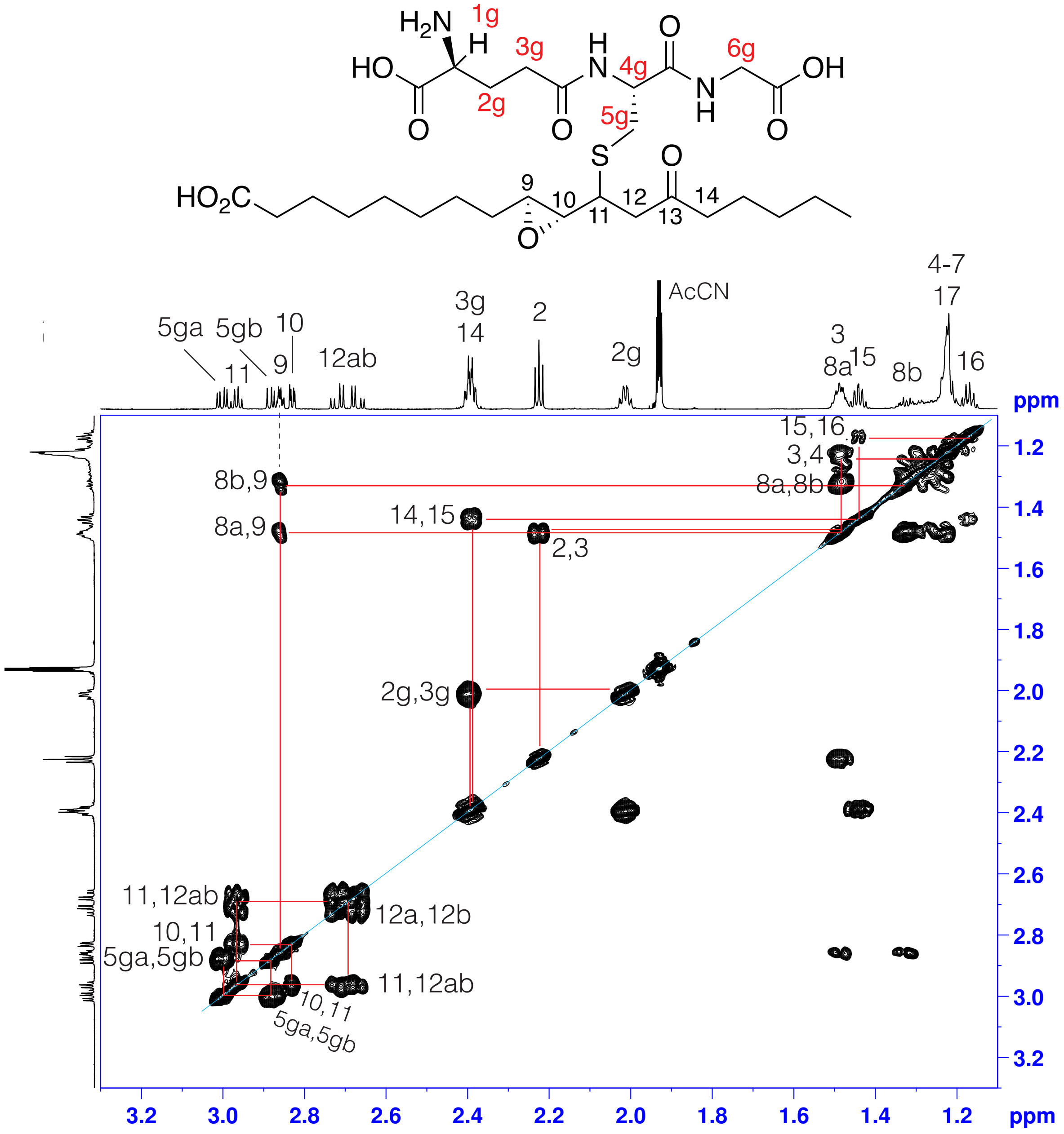

Supplemental Figure S2: Partial ^{1}H-NMR spectrum (800 MHz, 1.1 – 3.3 ppm) and COSY analysis of a GSH adduct of 1a (9*S*,10*S*-trans-epoxy-11*E*-13-oxo-octadecenoic acid)

The spectrum was recorded on the first-eluting diastereomer from RP-HPLC (cf. Fig. 1) in CD_3CN/D_2O (60:40 by volume). Upon thiol adduction, due to the higher priority of sulfur on the 11-carbon, the assignment of H10 changes to 10*R*. Not included in this partial spectrum are protons from the glutathione moiety further downfield: 1g, a triplet at 3.58 ppm; 6g(ab), two doublets centered on 3.72 ppm; and the 4g, a double of doublets at 4.46 ppm; and also included, the terminal methyl group at 0.81 ppm. The full 1D NMR spectrum is shown in the main text, Figure 2.

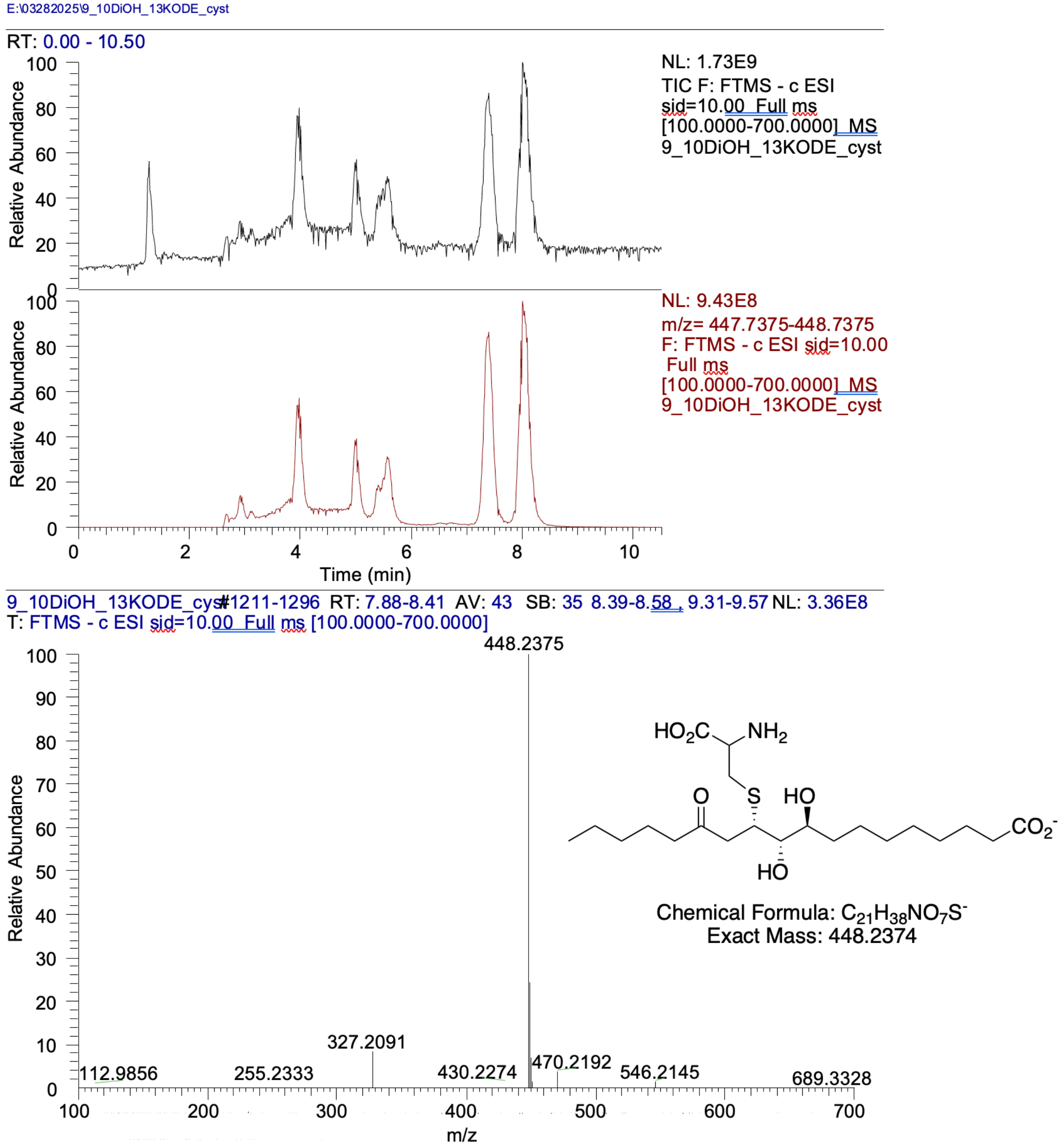

Supplemental Figure S3: LC-MS analysis of cysteine adducts of racemic 9,10-*erythro*-dihydroxy-11*E*-13-oxo-octadecenoic acid

L-Cysteine adducts to racemic dihydroxy-ketone in the 11*R* and 11*S* configuration on each enantiomer, giving four products in total. The two prominent peaks at 7.4 and 8.1 min are single stable species of adduct, one from each enantiomer, that chromatograph normally. The early-eluting diastereomers from each enantiomer are a mixture of interconverting species eluting at retention times between 3 and 6 minutes. (Similar chromatographic behavior is illustrated for the GSH adducts of a chiral dihydroxy-ketone in the main text, Figure 3). Top panel: Total Ion Current. Middle: profile of negative ion *m/z* 448, M-H$^-$. Lower panel: mass spectrum of the peak at retention time 8.1 min (*m/z* 448.23, M-H$^-$). The products were analyzed by LC-MS on a Kinetex 5 µ C18 column (100 x 3 mm) with a solvent of MeOH/H_2O-10 mM NH_4Ac, pH 5 at a flow rate of 0.3 ml/min. Mass spectra were recorded on a Thermo Q Exactive HF hybrid quadrupole/orbitrap scanning in negative ion ESI over a mass range of m/z 100 – 700.

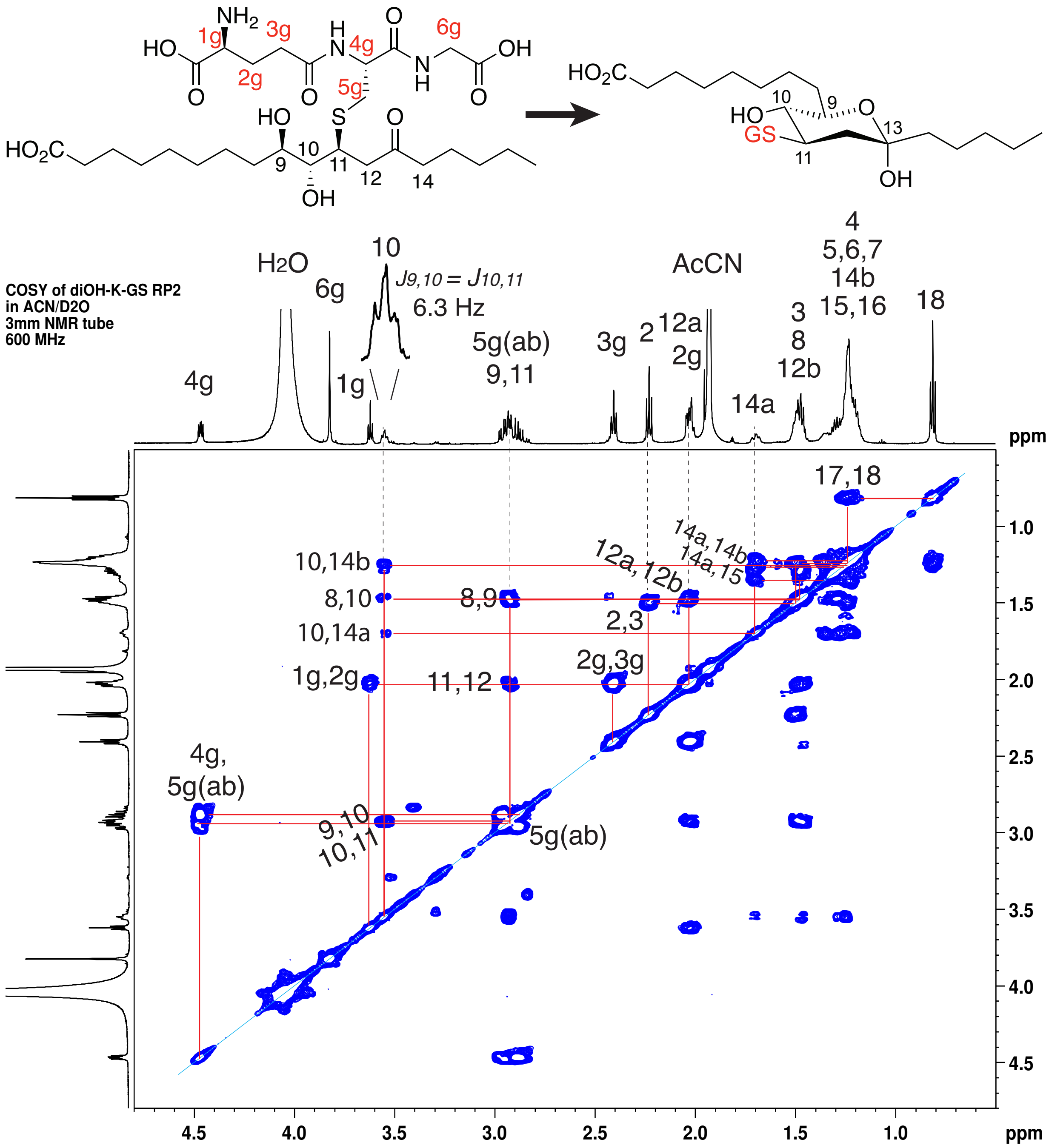

Supplemental Figure S4: ^{1}H-NMR spectrum (600 MHz) and COSY analyses of the glutathione conjugate of 2 (9*R*,10*S*-dihydroxy-11*E*-13-oxo-octadecanoic acid)
The spectrum and COSY of the second-eluting (RP-HPLC) diastereomeric conjugate of 9*R*,10*S*-dihydroxy-11*E*-13-oxo-octadecenoic acid recorded in CD_3CN/D_2O (60:40 by volume). Upon thiol adduction the higher priority of sulfur at C11 changes the C10 hydroxyl assignment to 10*R*. The main difference from the spectrum of the GSH conjugate of the enantiomeric dihydroxy-ketone is the signals from 5g(ab), H9 and H11 are a single cluster in the above spectrum (cf. Figure 4, main text). GS = Glutathione

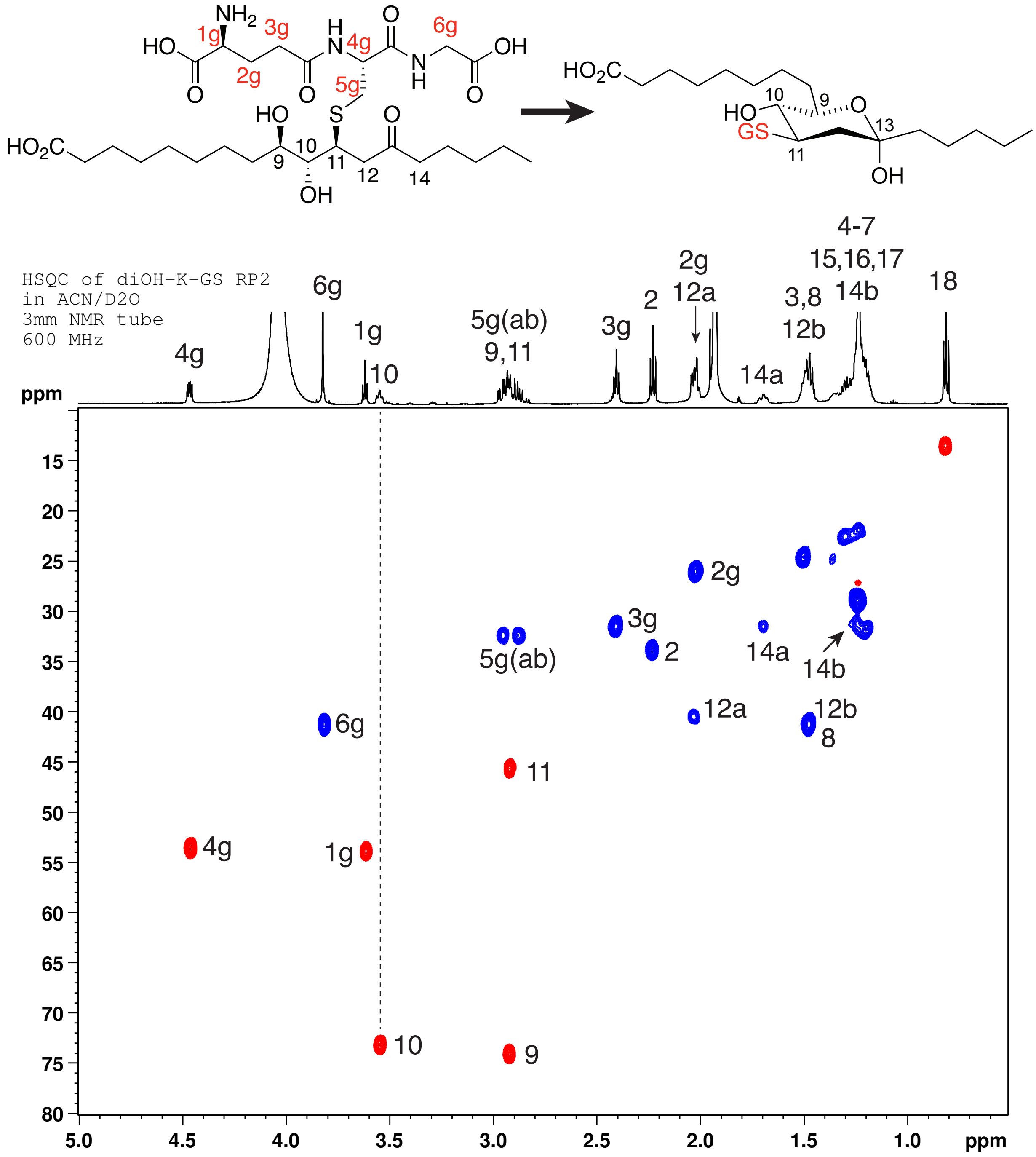

Supplemental Figure S5: HSQC spectrum of the glutathione conjugate of 2, (9*R*,10*S*-dihydroxy-11*E*-13-oxo-octadecenoic acid)

This is the multiplicity edited HSQC spectrum of the second-eluting (RP-HPLC) diastereomeric glutathione conjugate of 2, 9*R*,10*S*-dihydroxy-11*E*-13-oxo-octadecenoic acid, recorded in CD_3CN/D_2O (60:40 by volume). The COSY spectrum is shown in Supplemental Figure S4. GS = Glutathione.

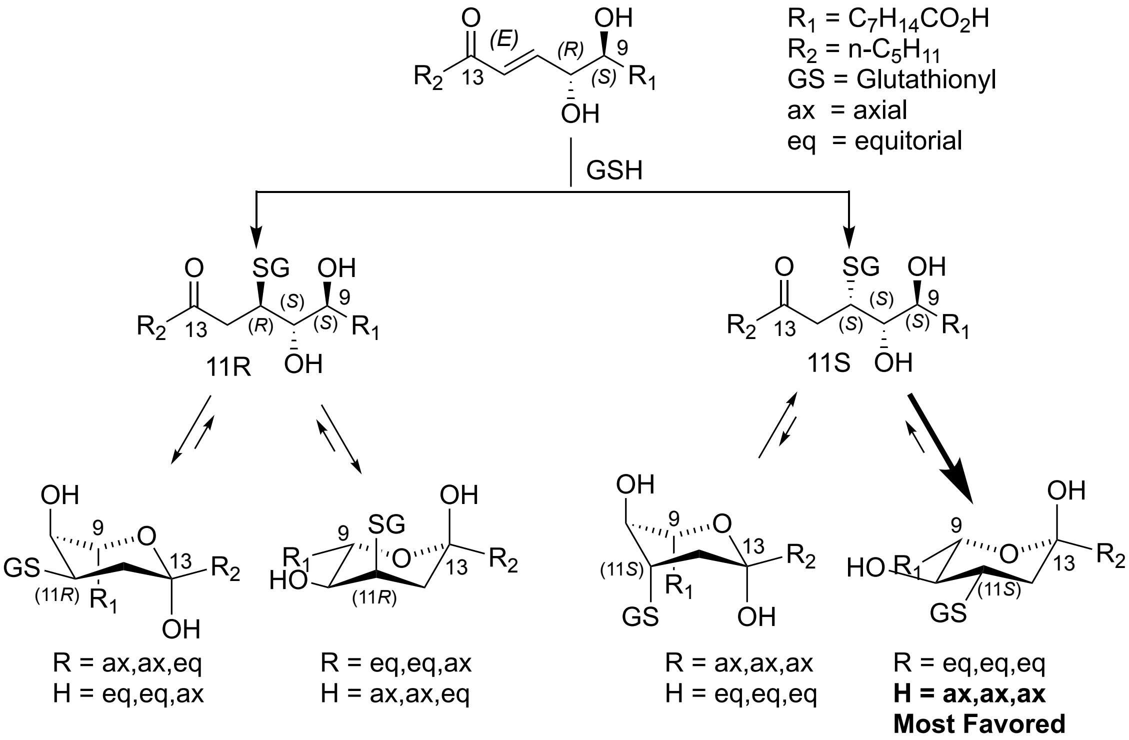

Supplemental Figure 6: Hemiketal formation upon glutathione adduction to 9*S*,10*R*-dihydroxy-ketone 2a

The 11*R* adducts (left hand side, and that appear as multiple peaks on RP-HPLC) exist as interconverting hemiketals. The most favored form of the 11*S* isomer (right hand structure) is the late-eluting and "stable" diastereomer on RP-HPLC and is deduced to have H9-H10-H11 in the axial/axial/axial configurations (Discussion, main text).
